# Multi‐scale imaging of α‐synuclein, dopamingeric neurodegeneration and microstructural changes in α‐synuclein PFF mouse model of Parkinson's disease

**DOI:** 10.1002/alz70855_097809

**Published:** 2025-12-23

**Authors:** Benjamin F. Combes, Alicia Rosenau, Sheng‐Fu Huang, Maria Karatsoli, Marco Reisert, Yi Chen, Hikari Yoshihara, Annika Keller, Dominik von Elverfeldt, Andras Jakab, Wolfgang H Oertel, Roger M. Nitsch, Fanni Geibl, Daniel Razansky, Martin Henrich, Christoph Hock, Ruiqing Ni

**Affiliations:** ^1^ University of Zurich, Zurich, Switzerland; ^2^ Philipps‐University Marburg, Marburg, Marburg, Germany; ^3^ University Hospital Zurich, Zurich, Zurich, Switzerland; ^4^ University of Zurich, Zurich, Zurich, Switzerland; ^5^ Universitätsklinikum Freiburg, Freiburg, Freiburg, Germany; ^6^ ETH Zurich, Zurich, Zurich, Switzerland; ^7^ University Hospital Zurich, Zurich, Switzerland; ^8^ Kinderspital Zürich, Zurich, Zurich, Switzerland; ^9^ Philipps University, Marburg, Germany; ^10^ ETH Zurich & University of Zurich, Zurich, Switzerland; ^11^ University of Zurich, 8001, Switzerland

## Abstract

**Background:**

The goal of this project is to map the spatial‐temporal association between α‐synuclein inclusions, dopaminergic neurodegeneration and white matter microstructural changes of a mouse model of α‐synucleinopathy

**Method:**

We injected the substantia nigra pars compacta (SNc) of wild‐type mice unilaterally with preformed‐fibrils (PFFs) or monomeric α‐synuclein, and performed at 12 and 20 weeks‐post‐injection (wpi) the *ex vivo* diffusion tensor imaging (DTI) magnetic resonance imaging (MRI) at 9.4 T to study white matter structural connectivity, and light‐sheet microscopy (LSM) to map the distribution of α‐synuclein aggregates (pS129) and dopaminergic neurodegeneration (tyrosine hydroxylase staining). Voxel‐based analysis (VBA) and atlas‐based analysis (ABA) of DTI mouse brain data were performed and validated by immunostaining.

**Result:**

Injection of PFFs led to a dense α‐synuclein pathology at 12 and 20 wpi in the dopaminergic neurons of the SNc, which was associated with neuronal cell death. This model also showed that the pathogenic α‐synuclein exhibited prion‐like seeding activity by converting the physiological form of α‐synuclein to pathogenic aggregates and spread to other brain regions, mainly in the striatum as shown by the whole brain LSM. The loss of dopaminergic cells in the SNc was accompanied by dopaminergic denervation of the striatum. VBA showed a decrease in fractional anisotropy in the corpus callosum and internal capsule of PFFs‐ compared to monomers‐injected mice at 12 wpi. These data indicates that dopaminergic neurodegeneration is associated with structural connectivity changes by DTI MRI.

**Conclusion:**

The α‐synuclein PFF mouse model displayed abundant α‐synuclein pathology, white matter impairment, and dopaminergic neuronal cell death, especially in the nigrostriatal pathway as seen in patients with Parkinson's disease. The *ex vivo* MRI‐LSM platform is an ideal tool for understanding the spreading of pathologies at circuit and whole brain levels.

